# Measures of Utility Among Studies of Genomic Medicine for Critically Ill Infants: A Systematic Review

**DOI:** 10.1001/jamanetworkopen.2022.25980

**Published:** 2022-08-10

**Authors:** Katharine Press Callahan, Rebecca Mueller, John Flibotte, Emily A. Largent, Chris Feudtner

**Affiliations:** 1 The Children’s Hospital of Philadelphia, Philadelphia, Pennsylvania; 2 Department of Medical Ethics and Health Policy, Perelman School of Medicine at the University of Pennsylvania, Philadelphia

## Abstract

**Importance:**

Genomic medicine holds promise to revolutionize care for critically ill infants by tailoring treatments for patients and providing additional prognostic information to families. However, measuring the utility of genomic medicine is not straightforward and has important clinical and ethical implications.

**Objective:**

To review the ways that researchers measure or neglect to measure the utility of genomic medicine for critically ill infants.

**Evidence Review:**

This systematic review included prospective full-text studies of genomic medicine of both whole exome and genome sequencing in critically ill infants younger than 1 year. PubMed, Embase, Scopus, and Cochrane Library databases, the Cochrane Database of Systematic Reviews, and the ClinicalTrials.gov register were searched with an English language restriction for articles published from the inception of each database through May 2022. Search terms included variations of the following: *gene*, *sequencing*, *intensive care*, *critical care*, and *infant*. From the included articles, information on how utility was defined and measured was extracted and synthesized. Information was also extracted from patient cases that authors highlighted by providing additional information. Spearman rank-order correlation was used to evaluate the association between study size and utility.

**Findings:**

Synthesized data from the 21 included studies reflected results from 1654 patients. A mean of 46% (range, 15%-72%) of patients had a positive genetic test result, and a mean of 37% (range, 13%-61%) met the criteria for experiencing utility. Despite heterogeneity in how studies measured and reported utility, a standardized framework was created with 5 categories of utility: treatment change, redirection of care, prognostic information, reproductive information, and screening or subspecialty referral. Most studies omitted important categories of utility, notably personal utility (patient-reported benefits) (20 studies [95%]), utility of negative or uncertain results (15 [71%]), and disutility (harms) (20 [95%]). Studies disproportionally highlighted patient cases that resulted in treatment change. Larger studies reported substantially lower utility (*r* = −0.65; *P* = .002).

**Conclusions and Relevance:**

This systematic review found that genomic medicine offered various categories of utility for a substantial proportion of critically ill infants. Studies measured utility in heterogeneous ways and focused more on documenting change than assessing meaningful benefit. Authors’ decisions about which cases to highlight suggest that some categories of utility may be more important than others. A more complete definition of utility that is used consistently may improve understanding of potential benefits and harms of genetic medicine.

## Key Points

**Question:**

How do studies of genomic medicine in critically ill infants measure its utility?

**Findings:**

In this systematic review of 21 studies including 1654 infants, utility was heterogeneously measured and reported but generally fit into 5 categories: treatment change, redirection of care, prognostic information, reproductive information, and screening or subspecialty referral. Measurement of utility was inconsistent, focused on documenting change rather than assessing meaningful benefit, and omitted patient-reported benefits, utility of negative or uncertain results, and disutility (harms).

**Meaning:**

The findings suggest a need for a complete, broadly accepted, and consistently applied definition of utility for genomic medicine.

## Teaser

This systematic review examines how studies of genomic medicine among critically ill infants measure and report the utility of genetic testing.

## Introduction

Genetic sequencing is becoming more efficient and less expensive, making genomic medicine (whole exome and genome sequencing and associated care) increasingly available in clinical practice.^1^ Given the high rates of genetic disease among critically ill infants, use of genomic medicine in this population is considered a breakthrough application, and the demonstrated utility of genomic medicine—generally understood as the likelihood that a genetic test will result in improved outcomes—among critically ill infants may prompt its incorporation into medical practice more broadly.^2^ Genomic medicine holds promise to revolutionize clinical care by simplifying the diagnostic process,^3^ improving treatments,^4^ providing families with answers and information about the future,^5^ and, when the prognosis is grim, shifting the focus to palliative care.^6^ Nevertheless, there is not consensus on how to best assess genomic medicine’s utility in this important population.^7,8^ How researchers measure or neglect to measure utility has important clinical and ethical implications.

The potential utility of genomic medicine encompasses direct effects on patient care as well as knowledge for both families and physicians.^7^ However, because genomic medicine can lead to a broad range of findings with an even broader array of effects on care, the utility of genomic medicine cannot be tied to a single, easily measurable health outcome.^9^ Commonly ascertained outcomes for other medical interventions, such as length of hospital stay or quality-adjusted life-years, are difficult to apply in the genomic medicine context because the aim of genomic medicine is not always to reduce the length of hospital stay or to prolong life.^6^ Intermediate outcomes, such as diagnostic yield, or process measures, such as changes in care, are poor proxies for utility.^6,7,10^ Furthermore, personal utility or patient-endorsed benefits can be subjective and may include the psychological value of having an explanation for a disease and a clearer sense of the future.^8,11,12,13^ Additional considerations for assessing the utility of genomic medicine include the effects of negative or uncertain results and secondary findings (ie, incidental genetic diagnoses unrelated to the reason for testing).^14,15,16^

Although the complexity and subjectivity of measuring utility are recognized, there have been efforts to evaluate different benefits associated with genomic medicine, including from the perspectives of clinicians^17^ and families.^11,12^ Conceptual frameworks of utility for genomic medicine that incorporate potential benefits and harms for the individual, the family, and society have previously been proposed,^7,9,18^ and an 18-item clinician-reported genetic testing utility index that assesses diagnosis, management, and familial and psychosocial impact has been developed.^19^ However, little is known about how utility is measured in clinical studies, which are the types of studies that aim to demonstrate utility. Although several reviews combined measurements of utility between studies, they took reported utility at face value and did not assess or compare the utility measures used.^20,21,22^ In this study, we aimed to investigate the measurement and components of utility in studies of genomic medicine in critically ill infants, to assess current gaps in measurement, and to suggest steps to improve assessment of utility in the future.

## Methods

This systematic review followed the Preferred Reporting Items for Systematic Reviews and Meta-analyses (PRISMA) reporting guideline for the literature search.^23^ Because this study used previously published, deidentified data, the Children’s Hospital of Philadelphia institutional review board deemed it exempt. A librarian assisted us in a literature search of the PubMed, Embase, Scopus, and Cochrane Library databases, the Cochrane Database of Systematic Reviews, and the ClinicalTrials.gov register for articles published from the inception of each database through May 2022, focusing on use of genomic medicine in infants younger than 1 year. To capture all studies of genomic medicine in critically ill infants, we included the following keywords or Medical Subject Headings terms across all databases: (sequenc* OR *sequencing* OR screen* OR test* OR *analysis*) AND (*gene* OR *genes* OR *genetic* OR *genomic* OR *genome* OR *nucleotide* OR *exome* OR *exomes* OR *whole-exome* OR *DNA*) AND (*critically ill* OR *critical illness* OR *critical care* OR *intensive care*) AND (infan* OR neonat* OR newborn*). We limited the searches to English language. We also reviewed the reference lists of all relevant articles and of review articles from the past 5 years that examined the utility of genomic medicine among infants.^20,21,22,24^ We imported all results into Zotero reference management software, version 6.0.9 (Corporation for Digital Scholarship), for deduplication and title and abstract review. We included full-text studies that met the following criteria: prospective studies of genomic medicine in patients younger than 1 year who were hospitalized in any intensive care unit. We excluded studies that included only patients with a specific symptom (eg, encephalopathy).

Two coders (K.P.C. and R.M.) reviewed each article that met the inclusion criteria. First, both coders independently recorded the way that each study divided utility (ie, improvement in outcomes) into categories or types. Together, the coders then created a coding framework that represented the most common, mutually exclusive categories of utility. Next, the coders independently reviewed each article again, extracted quantitative data about the number of patients who experienced utility as defined by the authors of the respective studies, and aligned these data with the categories of utility from the coding framework. The coders included reported utility from both positive and negative results. They also recorded the methods used to assess utility, whether the potential utility of negative results was discussed, and how studies dealt with secondary findings. In addition, the coders assessed whether studies highlighted specific patient cases to illustrate or serve as exemplars of utility. If so, the coders characterized highlighted cases and the categories of utility into which they fit. Articles were coded twice (ie, once by each coder), and discrepancies were resolved by consensus. Throughout this process, the coders logged and discussed qualitative notes about ways in which each article conformed to or deviated from the categories of the coding framework.

### Statistical Analysis

We stored the data in an Excel, version 2021 (Microsoft) workbook and analyzed the data using Stata, version 17.1 (StataCorp LLC). We calculated the proportion of patients who experienced each category of utility by comparing the number of patients in that category with the total number of patients who experienced any utility. We compared representation of utility categories between the overall synthesized data and the subset of highlighted patient cases. We used Spearman rank-order correlation to evaluate the association between study size and utility with a 2-sided α level of .05.

## Results

**Figure 1.**
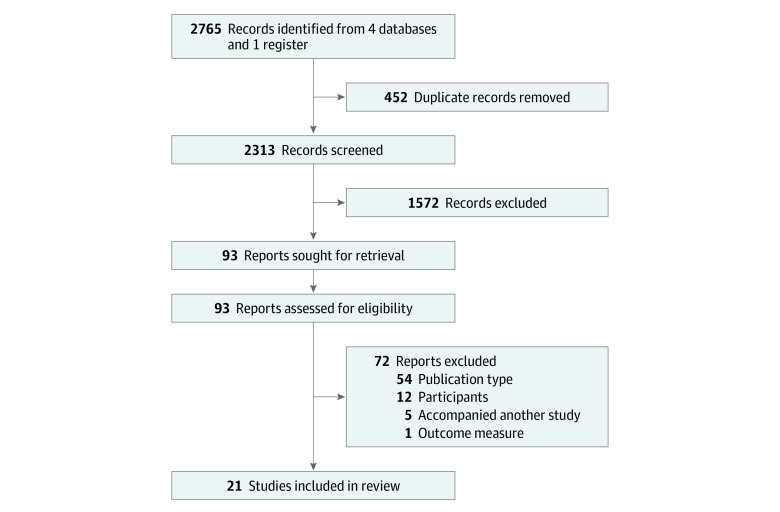
Study Screening and Selection Process

Twenty-two articles met our eligibility criteria (Figure 1).^25,26,27,28,29,30,31,32,33,34,35,36,37,38,39,40,41,42,43,44,45,46^ All but 1 study^46^ included some measure of utility (although authors used different terms for this concept, such as *usefulness*^35^ or *impact on medical decision-making*^31^) and therefore were included in subsequent analysis. Included studies reflected results from a total of 1654 patients. Most studies (18 [86%])^26,27,28,29,30,32,33,34,35,36,37,39,40,41,42,43,44,45^ took place in both neonatal and pediatric intensive care units, and inclusion criteria for participants ranged from specific (eg, a suspected known monogenic disorder) to broad (eg, unknown etiology of disease) (eTable in the Supplement). The studies contained a mean of 79 patient cases (range, 7-354 patient cases). A mean of 46% (range, 15%-72%) of patients had a positive genomic test result, and a mean of 37% (range, 13%-61%) were reported to have experienced utility (eTable in the Supplement). Larger studies reported substantially lower utility (*r* = −0.65; *P* = .002) (eFigure in the Supplement).

Ten of the studies (48%) reported how patient cases were assessed for potential utility.^26,30,31,33,35,38,39,40,41,43^ When reported, the most common method of assessment was discussion with a treating physician (11 studies [52%]).^26,30,31,33,35,38,39,40,41,42,43^ Some studies additionally incorporated medical record review by 1 (4 studies [19%])^30,31,38,42^ or more (2 studies [10%])^35,43^ team members to confirm utility. One study (5%)^40^ included a parent questionnaire.

Although most studies (15 [71%])^25,26,27,28,29,30,31,32,33,34,36,37,41,42,43^ limited assessment for potential utility to patients with positive genetic test results, 6 studies (29%) also assessed patients with negative results using the same methods.^35,38,39,40,41,43^ No studies specifically addressed the effects of uncertain genetic findings; however, 2 studies (10%)^32,34^ specified that they excluded uncertain genetic variants from result reports. Studies also differed in whether secondary findings were reported and, correspondingly, whether such findings were assessed for potential utility. In 9 studies (43%), secondary findings were reported to families,^27,28,29,35,36,40,41,43,44^ whereas in 6 studies (29%), they were not^25,30,31,32,33,38^; the remaining 6 studies (29%) provided no information about management of secondary findings.^26,34,37,39,42,45^

The coders identified 5 common categories of utility, which were included in the coding framework: (1) treatment change (deviation from or revisiting of the active care plan, such as a change in medication), (2) redirection of care (adoption of a palliative care plan, usually with death occurring during the hospitalization), (3) screening or referral (recommendation for a new screening examination or subspecialty referral), (4) prognostic information (information about the infants’ prognoses), and (5) reproductive information (information about the risk of recurrence in subsequent children, provided to the infants’ parents). Four studies (19%) assessed all 5 of these categories of utility,^31,36,38,40^ 13 (62%) assessed 3 or 4 categories,^25,26,27,29,30,32,33,34,35,39,42,43,44^ and the remaining 4 (19%) assessed 1 or 2 categories^28,37,41,45^ (eTable in the Supplement).

Even when studies assessed the same category of utility, investigators used different criteria to assign patient cases to the category. For instance, Elliott et al^31^ recorded affirmation of a current medication as a treatment change, whereas Wang et al^37^ counted only new treatments in this category.

**Figure 2.**
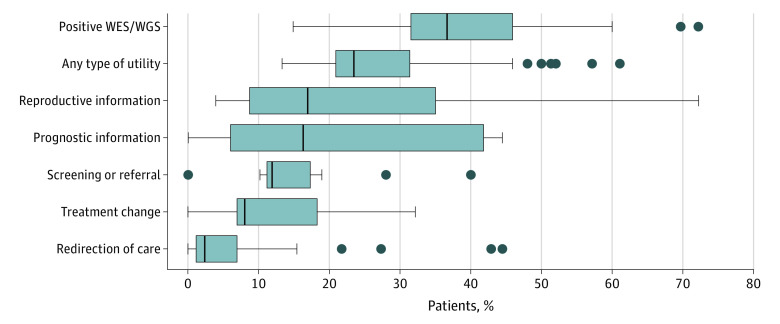
Comparison of Utility Categories The vertical bar in each box plot represents the median value for the outcome of interest; box edges, the first and third quartiles; and box width, the IQR. Whiskers extend to the smallest and largest observations within 1.5 times the IQR of the quartiles. Dots beyond the whiskers represent point estimates for studies that were outliers. WES indicates whole exome sequencing; WGS, whole genome sequencing.

**Figure 3.**
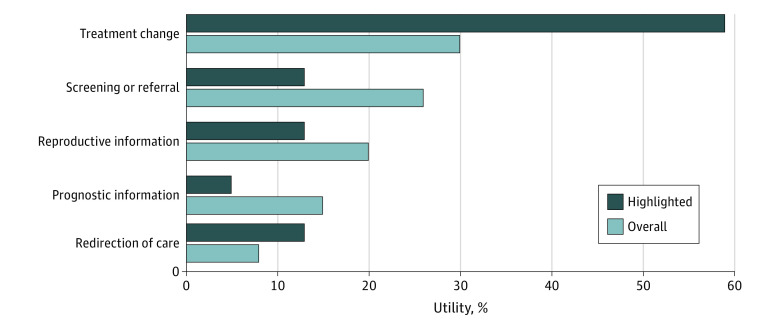
Comparison of Overall vs Highlighted Utility

Comparing the 21 studies, genetic results provided reproductive information in 27% of pooled patient cases (range of cases per study, 4%-72%), provided prognostic information for 16% (range per study, 0%-44%), and led to a subspecialty referral or additional screening for 15% (range per study, 0%-40%) (Figure 2 and eTable in the Supplement). Testing led to treatment change for 14% (range, 0%-32%) of patients and to redirection toward palliative care for 14% (range, 0%-44%). Eleven studies (52%) highlighted exemplary cases of utility (Figure 3).^25,26,27,29,31,32,38,39,42,43,44^ Treatment changes were overrepresented in this subset compared with overall cases (59% vs 30%). Prognostic information and screening or referral were underrepresented in the highlighted subset compared with overall cases (prognostic information: 5% vs 15%; screening or referral: 13% vs 26%). Most studies omitted important categories of utility, notably personal utility (patient-reported benefits) (20 studies [95%]), utility of negative or uncertain results (15 [71%]), and disutility (harms) (20 [95%]).

## Discussion

We reviewed prospective studies of genomic medicine in critically ill infants to evaluate the ways that these studies defined and measured utility. Researchers appeared to give importance to measuring and reporting utility because most studies included some measure of utility. Assessment of utility is appropriate because the goal of clinical genetic testing of critically ill infants is to benefit patients and families. A substantial proportion of patients were reported to experience some category of utility. Informational utility (ie, provision of prognostic and reproductive information) was more commonly reported than utility related to action (ie, treatment changes and redirection of care).

The ways in which researchers defined, measured, and reported utility varied. The broad range of the reported utility overall and particularly for the categories of reproductive and prognostic information resulted in part from heterogeneous assessment criteria and methods. The inverse correlation between sample size and the proportion of patients who experienced utility was likely also the result of methodologic heterogeneity. Smaller studies can more feasibly conduct in-depth clinician interviews or medical record reviews and thereby may have detected more effects that could be recorded as utility. Publication bias also likely contributed to this correlation because larger studies are more likely to be published independent of their findings. Half of the studies (10 [48%]) omitted their methods of assessing utility. Although this may in part reflect variation in study focus, the omission may also demonstrate the absence of clear standards in this research area and limited our ability to compare results and understand heterogeneity among studies.

Despite this variability, the studies shared common shortcomings. First, each of the 5 broad categories of utility was given equal weight, which may not be appropriate. The disproportionate highlighting of certain categories of utility, particularly treatment changes, suggests the authors valued some categories more than others. Because the frequency of cases in each of the 5 broad categories of utility differed, assigning categories different weights would affect assessments of utility overall. If treatment changes are considered to be of greater worth, as suggested by the analysis of highlighted cases, the current practice of equal weighting of utility categories would result in an overestimation of utility. Research using the Delphi method confirms that clinicians unequally weigh different types of utility,^17^ but this was not reflected in the methods used by the studies in this review.

A related concern is that studies did not adequately differentiate within single categories of utility. For example, changes in patient care did not necessarily correlate with benefit as perceived by patients, families, or physicians and should be considered a surrogate rather than primary marker of benefit.^7,47^ Only the studies by Dimmock et al^40,41^ and Kamolvisit et al^45^ attempted to record whether the change was expected to benefit the patient and family. Of note, Dimmock et al^41^ reported that with 1 exception, “changes in longer-term outcomes were deemed too speculative to be confident about cost savings or improved quality of life beyond the initial episode of care.” To make this more concrete, if 1 diagnosis informs prescribing of a new drug that largely alleviates the infant’s symptoms and another diagnosis affirms continuation of a drug that only moderately decreases symptoms, these changes are recorded as equally beneficial in currently used measures of utility; however, most physicians and families would likely value the former more highly than the latter. Although changes in care may be a useful surrogate marker of benefit, measurement of utility for neonatal genomic medicine should include the magnitude of that benefit.^48^

An additional shortcoming is that the studies included in this review largely neglected 3 important categories of utility and disutility highlighted elsewhere in the literature: (1) personal utility and families’ perspectives, (2) negative and uncertain results and secondary diagnoses, and (3) potential disutility or harmful effects.^9,11,12,13,49,50,51,52,53,54^ Although personal utility is ranked among the most important benefits of genomic medicine by clinicians,^17^ only 1 study^40^ included data from parents or any measure of personal utility.^40,55^ Future approaches to assessing the utility of genomic medicine should seek families’ direct input.

Negative and uncertain results and secondary diagnoses were also largely disregarded despite a growing body of research showing that uncertain and negative genetic test results have consequences for clinicians and patients.^49,50,51,52^ Moreover, because most genetic test results are negative or uncertain, disregarding them is a substantial oversight. Secondary diagnoses were also inconsistently reported to families and thus inconsistently assessed for utility. This is problematic because secondary diagnoses, particularly the identification of adult-onset conditions in infancy, have the potential to both benefit and harm patients and families.^56,57^ In addition, study practices often deviate from the recommendation of the American College of Medical Genetics to report a curated list of secondary findings on exome and genome sequencing (regarded as a best practice); thus, the application of the study findings to actual practice is limited.^15^

Assessment of potential disutility (ie, harms) from the genetic results were reported in only 1 study.^40^ Since the introduction of genomic medicine into clinical practice, there has been acknowledgment of potential benefits and harms of this technology.^9,13,53,54^ In most studies in this review, however, any potential confusion for clinicians or parents, misuse of genetic results, or bias applied in interpreting results was unmeasured. Prior work has empirically substantiated the possibility that neonatologists may misapply genetic information that is uncertain or portends future disability.^52^ In this review, none of the studies addressed concerns from the disability community about the use of genetic tests to discriminate against patients with disabilities.^58^ Although many studies cited cases in which families elected palliative care based on genetic results, the studies did not describe the extent of associated disability or whether parents received counseling about disability. Readers therefore cannot judge the extent to which these data might reflect ableism.^52,58^ Going forward, given the prognostic uncertainty and range of phenotypes associated with many genetic conditions, a better understanding of how prognoses are framed and their psychosocial effects on families is worth exploring.^59^

More completely capturing the utility of genomic medicine will require a directed effort to integrate the growing literature on utility into studies of genomic medicine in neonates as well as new efforts to capture the perspectives of various stakeholders.^9^ To begin, researchers should standardize measurement of utility using thoughtful extant frameworks, such as the Clinician-reported Genetic Testing Utility Index by Hayeems et al,^19^ which is currently being adapted to the neonatal context. Efforts are ongoing to integrate economic measures and the perspective of payers into utility.^18,41^ Longer-term data on the effects of genomic medicine for critically ill infants will inform more nuanced estimates of benefit. Comprehensive, standardized data are difficult to collect but also critical for refining testing indications and educating both parents and clinicians about the benefits and risks of genomic medicine.

### Limitations

This study has limitations. First, although we attempted to decrease the heterogeneity of included studies by excluding retrospective studies and those focused on a single symptom (eg, encephalopathy), we synthesized data from distinct studies that used different genetic technologies, testing indications, and methods for evaluating utility. Second, because we synthesized data from studies that we suggest inadequately captured utility and omitted important types of utility, the synthesized data cannot fully relay the value of genomic medicine. Given the strong correlation between sample size and utility, the synthesized data also likely reflected publication bias. Therefore, we have resisted making overarching conclusions about the utility of genomic medicine for critically ill infants.

## Conclusions

The goal of genomic medicine for critically ill infants is to improve outcomes. Collectively, the studies in this systematic review revealed the potential utility of genomic medicine for a substantial proportion of these patients; however, the studies also revealed variability and shortcomings in how utility is measured in this patient population. Some researchers have hypothesized that genomic medicine’s “critical application” will be in the infant population, providing evidence of benefits that prompts adoption of genomic medicine in clinical practice more broadly.^60^ Clinicians, patients, families, and researchers may benefit from a complete, broadly accepted, and consistently applied definition of utility for genomic medicine.

## Notes

Supplement. **eTable.** Included Studies and Extracted Results **eFigure.** Association Between Sample Size and Utility
eReferences
