# Supplementary material for: Measures of Utility Among Studies of Genomic Medicine for Critically Ill Infants: A Systematic Review
Source: JAMA Netw Open. 2022 Aug 10;5(8):e2225980. doi: 10.1001/jamanetworkopen.2022.25980 (PMC9366540; doi:10.1001/jamanetworkopen.2022.25980)
Supplement: Supplement. — eTable. Included Studies and Extracted Results eFigure. Association Between Sample Size and Utility eReferences [file jamanetwopen-e2225980-s001.pdf]

Supplementary Online Content

Callahan KP, Mueller R, Flibotte J, Largent EA, Feudtner C. Measures of utility among studies of genomic medicine for critically ill infants: a systematic review. *JAMA Netw Open*. 2022;5(8):e2225980. doi:10.1001/jamanetworkopen.2022.25980

eTable. Included Studies and Extracted Results
eFigure. Association Between Sample Size and Utility
eReferences

This supplementary material has been provided by the authors to give readers additional information about their work.

© 2022 Callahan KP et al. *JAMA Network Open*.

Supplemental Material

eTable. Included Studies and Extracted Results

Counts represent number of reported patient cases that align with each category of utility.

First Author	Year	Setting	Inclusion Criteria	Patients Tested	Patients with Positive Results	Result had Utility	Result Changed Treatment	Result Led to Screening/ Subspecialty Referral	Results Informed Reproductive Counseling	Results informed Prognosis	Results Led to Redirection of Care
**Daoud**[1]	2016	NICU	Genetics consult	20	8	8	2	.	2	2	2
**Stark**[2]	2016	NICU, PICU, other inpatient unit	Suspected known monogenic disorder	80	46	15	8	10	28	.	.
**Bourchany**[3]	2017	NICU, PICU, prenatal clinic	Suspected genetic etiology	14	6	6	0	0	6	2	.
**Van Diemen**[4]	2017	NICU, PICU	Specified symptom patterns	23	7	5	.	.	2	.	5
Mestek-Boukhibar[5]	2018	NICU, PICU	Suspected genetic etiology, diagnosis expected to alter management	18	8	8	2	.	8	8	0
Petrikin[6]	2018	NICU, PICU	Suspected genetic etiology or unusual therapy response	37	12	11	4	7	9	.	.

© 2022 Callahan KP et al. *JAMA Network Open*.

**Elliott**[7]	2019	NICU	Specified symptom patterns	25	15	13	2	7	1	3	2
**French**[8]	2019	NICU, PICU	Suspected monogenic etiology	128	19	17	1	13	5	.	3
**Sanford**[9]	2019	NICU, PICU	Suspected monogenic etiology	7	4	4	1	0	.	.	3
**Wu**[10]	2019	NICU, PICU	Suspected genetic etiology	28	14	14	9	.	3	3	2
**Freed**[12]	2020	NICU, PICU	Suspected genetic etiology per committee review*	43	21	17	13	8	.	2	3
**Smigiel**[11]	2020	NICU, PICU	Specified symptom patterns	18	13	11	1	2	13	0	8
**Wang**[13]	2020	NICU, PICU	Suspected genetic etiology per geneticist review	33	23	10	10	.	.	.	9
**Gubbels**[14]	2020	NICU	Specified symptom patterns	50	29	24	7	20	17	3	6
**AGHAACF**[15]	2020	NICU, PICU	Genetics consult	91	49	35	1	14	.	.	14
**Dimmock**[15]	2020	NICU, PICU	Suspected genetic etiology or unusual therapy response	201	45	42	21	14	34	84	2

© 2022 Callahan KP et al. *JAMA Network Open*.

**Wu**[17]	2021	NICU, PICU	Suspected genetic etiology	202	74	38	13	24	.	.	7
**Dimmock**[18]	2021	NICU, PICU	Unknown etiology or unusual therapy response	184	74	58	56	.	.	.	.
**Krantz**[19]	2021	NICU, PICU	Suspected genetic disease	354	111	83	24	39	.	.	4
**Scholz**[20]	2021	NICU, PICU	Unknown etiology	61	28	28	8	11	28	3	.
**Kamolvisit**[21]	2021	NICU, PICU, adult ICU	Unknown etiology	37	20	19	8	.	.	.	.

© 2022 Callahan KP et al. *JAMA Network Open*.

eFigure. Association Between Sample Size and Utility

Each point represents a study and the fitted line represents a fractional polynomial model.

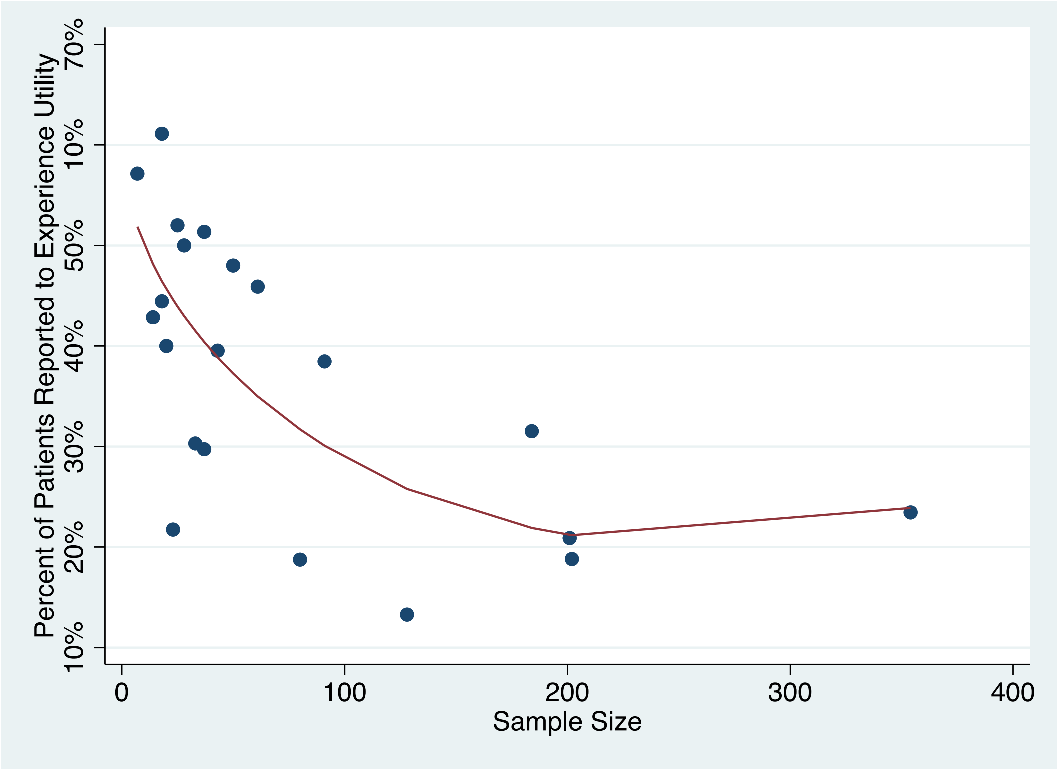

© 2022 Callahan KP et al. *JAMA Network Open*.

eReferences

1. Daoud H, Luco SM, Li R, et al. Next-generation sequencing for diagnosis of rare diseases in the neonatal intensive care unit. *CMAJ*. 2016;188(11):E254-E260. doi:10.1503/cmaj.150823

2. Stark Z, Tan TY, Chong B, et al. A prospective evaluation of whole-exome sequencing as a first-tier molecular test in infants with suspected monogenic disorders. *Genet Med*. 2016;18(11):1090-1096. doi:10.1038/gim.2016.1

3. Bourchany A, Thauvin-Robinet C, Lehalle D, et al. Reducing diagnostic turnaround times of exome sequencing for families requiring timely diagnoses. *Eur J Med Genet*. 2017;60(11):595-604. doi:10.1016/j.ejmg.2017.08.011

4. van Diemen CC, Kerstjens-Frederikse WS, Bergman KA, et al. Rapid Targeted Genomics in Critically Ill Newborns. *Pediatrics*. 2017;140(4). doi:10.1542/peds.2016-2854

5. Mestek-Boukhibar L, Clement E, Jones WD, et al. Rapid Paediatric Sequencing (RaPS): comprehensive real-life workflow for rapid diagnosis of critically ill children. *J Med Genet*. 2018;55(11):721-728. doi:10.1136/jmedgenet-2018-105396

6. Petrikin JE, Cakici JA, Clark MM, et al. The NSIGHT1-randomized controlled trial: rapid whole-genome sequencing for accelerated etiologic diagnosis in critically ill infants. *npj Genomic Medicine*. 2018;3(1):1-11. doi:10.1038/s41525-018-0045-8

7. Elliott AM, du Souich C, Lehman A, et al. RAPIDOMICS: rapid genome-wide sequencing in a neonatal intensive care unit-successes and challenges. *Eur J Pediatr*. 2019;178(8):1207-1218. doi:10.1007/s00431-019-03399-4

8. French CE, Delon I, Dolling H, et al. Whole genome sequencing reveals that genetic conditions are frequent in intensively ill children. *Intensive Care Med*. 2019;45(5):627-636. doi:10.1007/s00134-019-05552-x

9. Sanford EF, Clark MM, Farnaes L, et al. Rapid whole genome sequencing has clinical utility in children in the pediatric intensive care unit. *Pediatr Crit Care Med*. 2019;20(11):1007-1020. doi:10.1097/PCC.0000000000002056

10. Wu ET, Hwu WL, Chien YH, et al. Critical Trio Exome Benefits In-Time Decision-Making for Pediatric Patients With Severe Illnesses. *Pediatr Crit Care Med*. 2019;20(11):1021-1026. doi:10.1097/PCC.0000000000002068

11. Smigiel R, Biela M, Szmyd K, et al. High impact of mitochondrial disorders in the critically ill infants hospitalized in the intensive care units diagnosed by RAPID WES. *J Inherit Metab Dis*. 2019;42((Smigiel R.; Biela M.; Bloch M.) Dep Paediatric and Rare Dis, Med Univ, Wroclaw, Poland):211. doi:10.1002/jimd.12153

12. Freed AS, Clowes Candadai SV, Sikes MC, et al. The Impact of Rapid Exome Sequencing on Medical Management of Critically Ill Children. *The Journal of Pediatrics*. 2020;226:202-212.e1. doi:10.1016/j.jpeds.2020.06.020

13. Wang H, Qian Y, Lu Y, et al. Clinical utility of 24-h rapid trio-exome sequencing for critically ill infants. *npj Genom Med*. 2020;5(1). doi:10.1038/s41525-020-0129-0

14. Gubbels CS, VanNoy GE, Madden JA, et al. Prospective, phenotype-driven selection of critically ill neonates for rapid exome sequencing is associated with high diagnostic yield. *Genetics in Medicine*. 2020;22(4):736-744. doi:10.1038/s41436-019-0708-6

© 2022 Callahan KP et al. *JAMA Network Open*.

15. Australian Genomics Health Alliance Acute Care Flagship, Lunke S, Eggers S, et al. Feasibility of Ultra-Rapid Exome Sequencing in Critically Ill Infants and Children With Suspected Monogenic Conditions in the Australian Public Health Care System. *JAMA*. 2020;323(24):2503-2511. doi:10.1001/jama.2020.7671

16. Dimmock DP, Clark MM, Gaughran M, et al. An RCT of Rapid Genomic Sequencing among Seriously Ill Infants Results in High Clinical Utility, Changes in Management, and Low Perceived Harm. *Am J Hum Genet*. 2020;107(5):942-952. doi:10.1016/j.ajhg.2020.10.003

17. Wu B, Kang W, Wang Y, et al. Application of Full-Spectrum Rapid Clinical Genome Sequencing Improves Diagnostic Rate and Clinical Outcomes in Critically Ill Infants in the China Neonatal Genomes Project. *Crit Care Med*. Published online 2021:1674-1683. doi:10.1097/CCM.0000000000005052

18. Dimmock D, Caylor S, Waldman B, et al. Project Baby Bear: Rapid precision care incorporating rWGS in 5 California children's hospitals demonstrates improved clinical outcomes and reduced costs of care. *Am J Hum Genet*. 2021;108(7):1231-1238. doi:10.1016/j.ajhg.2021.05.008

19. Krantz ID, Medne L, Weatherly JM, et al. Effect of Whole-Genome Sequencing on the Clinical Management of Acutely Ill Infants with Suspected Genetic Disease: A Randomized Clinical Trial. *JAMA Pediatr*. Published online 2021. doi:10.1001/jamapediatrics.2021.3496

20. Scholz T, Blohm ME, Kortüm F, et al. Whole-Exome Sequencing in Critically Ill Neonates and Infants: Diagnostic Yield and Predictability of Monogenic Diagnosis. *NEO*. 2021;118(4):454-461. doi:10.1159/000516890

21. Kamolvisit W, Phowthongkum P, Boonsimma P, et al. Rapid exome sequencing as the first-tier investigation for diagnosis of acutely and severely ill children and adults in Thailand. *Clin Genet*. 2021;100(1):100-105. doi:10.1111/cge.13963

© 2022 Callahan KP et al. *JAMA Network Open*.
